# A fully automated stress regional strain score as a prognostic marker of cardiovascular events in patients with normal CMR

**DOI:** 10.3389/fcvm.2023.1334553

**Published:** 2024-01-08

**Authors:** Théo Pezel, Solenn Toupin, Thomas Hovasse, Stéphane Champagne, Thierry Unterseeh, Teodora Chitiboi, Puneet Sharma, Francesca Sanguineti, Philippe Garot, Jérôme Garot

**Affiliations:** ^1^ Department of Cardiology, University Hospital of Lariboisiere, Université Paris-Cité, (Assistance Publique des Hôpitaux de Paris, AP-HP), Paris, France; ^2^ Inserm MASCOT-UMRS 942, Department of Data Science, University Hospital of Lariboisiere, Paris, France; ^3^ Department of Radiology, University Hospital of Lariboisiere, Université Paris-Cité, (Assistance Publique des Hôpitaux de Paris, AP-HP), Paris, France; ^4^ Cardiovascular Magnetic Resonance Laboratory, Institut Cardiovasculaire Paris Sud (ICPS), Hôpital Privé Jacques Cartier, Ramsay Santé, Massy, France; ^5^ MIRACL.ai laboratory, Multimodality Imaging for Research and Analysis Core Laboratory-Artificial Intelligence, Department of Data Science, Machine Learning and Artificial Intelligence in Health, University Hospital of Lariboisiere (AP-HP), Paris, France; ^6^ Siemens Healthcare France, Scientific Partnerships, Saint-Denis, France; ^7^ Siemens Healthcare GmbH, Department of CMR, Hamburg, Deutschland; ^8^ Digital Technologies and Innovation, Siemens Healthineers, Princeton, NJ, United States

**Keywords:** cardiac MRI (CMRI), strain, stress CMR, artificial intelligence, strain score, major adverse cardiac event (MACE)

Stress cardiovascular magnetic resonance (CMR) imaging has emerged as an accurate and cost-effective modality without ionizing radiation for risk stratification using ischemia and late gadolinium enhancement (LGE) as prognostic markers (1). Beyond these traditional stress CMR findings, recent studies have highlighted the incremental prognostic value of global longitudinal or circumferential strain using feature-tracking imaging measured during vasodilator stress (2), especially in patients with normal stress CMR, defined by the absence of ischemia and LGE, regardless of the LVEF value or the presence of wall motion abnormalities (3). Recent studies have shown that the regional circumferential myocardial strain (Ecc) could be a more accurate predictor of cardiovascular events than the LVEF value or global Ecc (4, 5). Also, it is suggested that an analysis of specific myocardial layers could further improve its prognostic value (4). The value of a stress CMR–derived layer-specific regional Ecc score compared with global Ecc has not yet been established. Therefore, in this short study, we aim to determine whether a fully automated regional circumferential strain score (RSS) measured during vasodilator stress CMR can provide incremental prognostic value in patients with normal CMR.

During the period between January and July 2017, we conducted a single-center study with a retrospective enrollment of all consecutive patients with normal stress CMR (Siemens Espree 1.5 T, absence of ischemia or LGE). A normal stress CMR examination was defined by the absence of both ischemia and LGE. Hyperemia was induced with dipyridamole (0.84 mg/kg over 3 min). A bolus of 0.1 mmol/kg gadolinium chelates was injected (5 ml/s) with acquisitions of four left ventricular short-axis and two long-axis views using first-pass perfusion imaging. Then, a stack of eight short-axis views covering the entire LV were obtained using the b-SSFP sequence. Cross-registered LGE images were acquired 10 min after contrast injection. An analysis of perfusion images was done visually by two blinded experienced operators. The definitions of ischemia and LGE were based on established criteria (1, 2). A fully automated machine learning algorithm was trained using a dense UNet on 3,000 CMR studies from the UK Biobank resource and validated on unseen CMR studies to assess the stress-GCS of each myocardial segment from short-axis cine images acquired at stress (3). All details of the AI software methodology have already been recorded (3). To calculate the RSS (4), each segment was rated from 0 to 2 points according to the Ecc value of each layer to grade the LV regional myocardial function for the 16-segment model as follows: (i) 0 points if Ecc was more than −10% for severe regional dysfunction; (ii) 1 point if Ecc was between −17 and −10% for moderate regional dysfunction; and (iii) 2 points if Ecc was <−17% for good regional function. Then, using endo-Ecc, mid-Ecc, and epi-Ecc for the 16-segment model, we defined Endo-, Mid-, and Epi-RSS, respectively, as three indices of segmental myocardial function ranging from 0 to 32 points. To summarize the overall regional myocardial function, we defined the RSS as a score ranging from 0 to 96 points, which corresponded to the sum of the Endo-, Mid-, and Epi-RSS. The follow-up consisted of yearly clinical visits and additional contacts in case of events. The primary outcome was the occurrence of MACE defined as cardiovascular mortality or nonfatal myocardial infarction. Cox regressions were used to evaluate the association of stress-RSS with the primary outcome after adjustment for the following traditional risk factors: age, male, body mass index, diabetes, hypertension, smoking, dyslipidemia, known coronary artery disease, and LVEF value.

**Figure 1:**
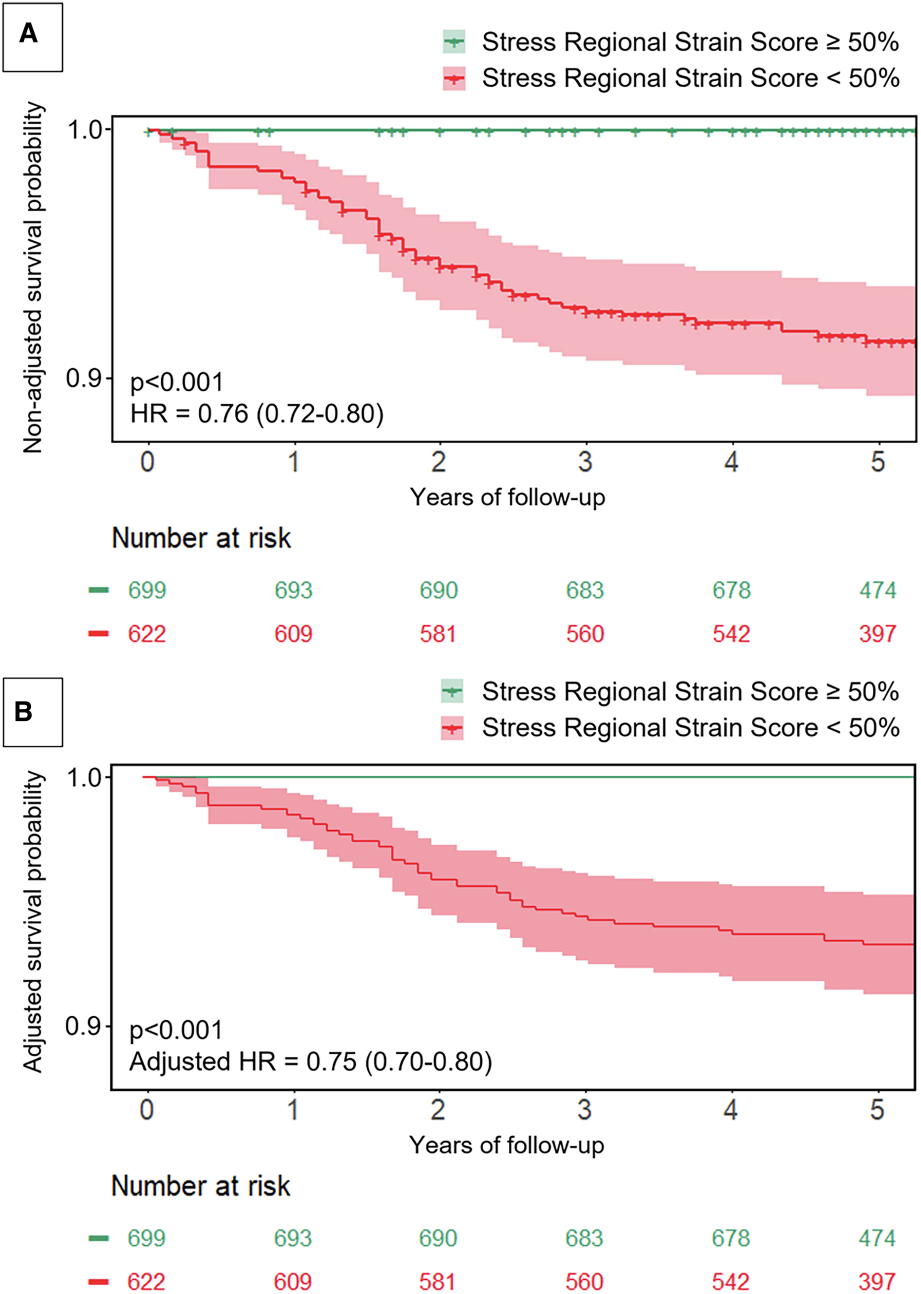
Survival curves for the occurrence of MACE according to RSS. The non-adjusted (**A**) and adjusted (**B**) event-free survival curves with confidence intervals for MACE were described. The adjustment used the final model including all traditional prognostic factors: age, male, body mass index, diabetes, hypertension, smoking, dyslipidemia, known coronary artery disease, LVEF value, and stress-RSS. HR indicates hazard ratio.

Among the 1,823 consecutive patients referred for stress CMR, 1,491 (82%) patients underwent a normal CMR examination (65 ± 12 years, 67% men). Among these, 1,321 (88.9%) completed clinical follow-up [median follow-up 5.1 (4.8–5.4) years] with 52 MACEs. After adjustment for traditional risk factors, stress-RSS was inversely associated with the occurrence of MACE [adjusted hazard ratio (HR), 0.75; 95% CI: 0.70–0.80, Figure 1]. The annual mortality rate was higher in patients with stress-RSS < 50% (6.3%/year) than in patients with stress-RSS value ≥ 50% (0%/year, *p* < 0.001). The addition of stress-RSS showed the best improvement in model discrimination and reclassification to predict MACE above traditional risk factors, including the LVEF value, and over stress CMR findings (C-statistic improvement: 0.29; NRI = 0.569; IDI = 0.153, Chi-2 global = 125, all *p* < 0.001; LR-test *p* < 0.001), corresponding to slightly better results than the stress-GCS (C-statistic improvement: 0.27; NRI = 0.539; IDI = 0.108, Chi-2 global = 98.9, LR-test *p* < 0.001).

This was a single-center study with high-volume stress CMR studies (>3,700/year) with a relatively low adverse-event rate. Baseline data and follow-up information on medications were not collected. These limitations were related to patient care and reflected current clinical practice. This retrospective study could not capture all confounding factors potentially affecting decision-making and patient risk. Finally, dipyridamole was used as a vasodilator agent mainly because of medico-economic reasons and its similar efficacy/safety profile to that of adenosine. Further randomized clinical trials are required to assess the prognostic impact of stress-RSS in patients with normal CMR.

In conclusion, this study shows that layer-specific regional Ecc measured during stress CMR (stress-RSS) provides a robust, independent, and incremental predictive value for MACE over traditional risk factors and stress CMR findings in patients with normal CMR. These findings might reflect the subtle functional consequences of the impairment of microvasculature not depicted by conventional (non-quantitative) stress CMR data.
